# Large-Scale Single Step Partial Purification of Potato Pectin Methylesterase that Enables the Use in Major Food Applications

**DOI:** 10.1007/s12010-014-1162-1

**Published:** 2014-08-27

**Authors:** Robin Eric Jacobus Spelbrink, Marco Luigi Federico Giuseppin

**Affiliations:** 1 AVEBE UA, 9607 PT Foxhol, The Netherlands; 2 AVEBEweg 1, 9607 PT Foxhol, The Netherlands

**Keywords:** Pectin methylesterase, Potato enzyme, Chromatography, Pectin gellation

## Abstract

Pectin methylesterase was extracted from potato tubers and partially purified in a single chromatographic step at large industrial scale. The preparation obtained in this way matched the temperature and pH profile of the species reported earlier by Puri et al. (*Food Chemistry 8*:203–213, 1982) and was enriched 23 times relative to the original potato tubers on a dry matter basis. Potato PME induced gel formation in calcium pectate across a broad pH range and should be suitable for application in the food industry. The procedure presented here represents a sustainable way to recover enzymes from vegetable juices.

## Introduction

Pectin methylesterase is one of the main cell wall modifying enzymes and occurs in plants and microorganisms. In the potato, it is important in strengthening the tubers texture during preparation [1].

Outside of its native context, pectin methylesterase (PME) is used in the food industry to induce gel formation in pectin solutions in distinct ways that depend upon the pectin’s composition. Pectin is not a completely homogenous molecule but consists of different regions that differ in composition [2]. One of these, the polygalacturonic acid region, occurs in either methylated or free carboxylic acid form. In the food industry, the methylated form of pectin is preferred for gelling in high-sugar systems such as jams and jellies. In this case, the low water activity decreases the pectins hydration, resulting in the formation of a gel.

The demethylated form of pectin can be crosslinked by calcium ions, thereby forming vast networks. This results in gel formation in systems that are low in dissolved solids.

When fruits and vegetables are processed, treatment by PME and calcium infusion can counteract the loss of structure that is caused by freezing. The PMEs that are commercially used for this purpose are exclusively derived from microorganisms.

Interestingly, a fundamental difference occurs between PMEs from fungal and vegetable sources in the pattern by which the enzyme demethylates its substrate [3] Plant-derived PMEs act in a blockwise manner, stripping the methyl groups from consecutive galacturonic acids, while fungal species act in a more random manner. These blocks of carboxylic acid groups are capable of forming a so-called egg-box structure upon interaction with calcium ions that results in stronger gels compared to gels that contain a more random pattern of demethylation [4]. This property makes plant PMEs a good choice for forming pectate gels. In addition, pectins with the egg-box structure are capable of interacting with- and stabilizing positively charged particles like casein in acidified dairy drinks [5].

The purification of plant PMEs has been described for a variety of species [6–8], including the potato [9, 10]. Several authors recently aimed to develop procedures for obtaining plant-derived PME for application in the food industry [11–13]. However, the pectin gels obtained via these preparations were not subjected to texture analysis in these studies. Although microbial PME preparations are on the market, no plant PME is currently commercially available. To ensure industrial applicability, such a preparation would have to match a series of requirements: PME activity should be high per unit of volume, detrimental side-activities and contaminating microorganisms should be minimized or removed, storage should be unproblematic and finally, the production costs should be sufficiently low to allow affordable price ranges for the final consumer product.

Recently, methods were developed to recover native protein from the side streams of the potato starch industry [14, 15]. Due to the mild nature of the recovery process, enzymes can be recovered in an active form. Since starch production for food applications is common and well developed, food safety and control of microorganisms is under tight control. Additionally, the use of potato as a raw material for PME preparations avoids undesirable side-activities since the potato tuber is inherently low in pectin-depolymerising enzymes: polygalacturonase has only been reported as the exo-enzyme and even the purified form has only a minimal effect on pectin viscosity [16]. The presence of pectolyase has not been reported in the potato tuber.

Surprisingly, the PME from potato tubers is recovered exclusively in a single fraction during industrial native protein chromatography. This PME has a pH and temperature profile identical to that described by Puri and coworkers [10] and shows inactivation behavior consistent with Anthon and Barrett [17]. Potato tuber PME induces gel formation in citrus pectin in the presence of calcium over a broad pH range.

The industrial chromatographic method described here should be applicable to other enzymes in vegetable juice, most prominently the potato lipase patatin.

## Experimentals

### Materials

Potato juice and potato protein concentrates were obtained from the potato protein factory at Gasselternijveen (AVEBE, the Netherlands). Citrus peel pectins for enzymatic analysis (P9311) were purchased from SigmaAldrich chemical company, commercial food-grade pectins were from CPKelco (Denmark).

### Methods

Experiments were performed at an ambient temperature of 21 °C ± 2 °C unless otherwise indicated. Protein concentrations were measured using a Sprint rapid protein analyser (CEM, North Carolina, USA) that was calibrated against the Kjeldahl method using a nitrogen conversion factor of 6.25.

### Chromatography

Protein concentrates were obtained from AVEBE/Solanic (Gasselternijveen, The Netherlands). These were produced in a continuous process essentially according to the procedure specified in EP1920662 [14]. Briefly, a continuous expanded bed (EBA) chromatography was performed by adjusting destarched potato juice to pH 6.0 and loading seven bed volumes onto CS174 EBA resin (Upfront Chromatography, Denmark) in upflow orientation. The bed was washed with 20-mM citrate buffer, pH 6.0, and eluted with 50 mM of NaOH (potato protein fraction 1), followed by a cleaning step with 1-M NaOH. The pH of the eluate was adjusted to 4.5 using acetic acid and lowered to 3.2 using hydrochloric acid. The protein fraction was concentrated by ultrafiltration to 20 % dry matter using a 10-kDa MWCO polyethersulfon membrane and stored frozen until use.

### Pectin Methylesterase Assay

Aliquots of potato extract containing PME were introduced into 50 mL of a 5-g/L pectin (SigmaAldrich P9311) solution that was maintained at pH-7.5 by a Metrohm pH-stat setup operating at ambient temperature. The consumption of 10.0 mM of NaOH was monitored over time at 1 min intervals over 10 min. Average consumption was determined via linear regression and expressed as units PME per milliliters liquid. Error bars in the figures and tables represent the standard error in the regression. One unit is defined as the quantity of enzyme that hydrolyzes 1 μmol/min of pectin under the conditions specified above. The pH-dependance of the preparation was determined by performing the assay at different pHs. For measurements at elevated temperatures, the reaction chamber was incubated in a water bath set and controlled to the desired temperature.

### Gellation Assay

Exploratory determinations of gel formation were performed using the method described by O’Brien et al. [18]. Briefly, a 10-g/L pectin solution (final concentration) was exposed to varying doses of potato protein concentrate that contained PME activity at different pHs between 3 and 8. By periodically tipping the vials containing the pectin solution, the time required to form a self-supporting gel could be estimated.

### PME Activity in Potato Juice Sampled at Different Times in the Campaign

Industrial potato processing typically takes place between the start of the potato harvest in late August and ends in late February when the last storage potatoes are processed. This period is referred to as a “campaign”. Since industrial starch potatoes represent a diverse range of cultivars that are grown on different soil types and that have seen different storage conditions, the protein composition and enzyme activities can vary. These properties were measured weekly in potato juice that was prepared form industrial starch potatoes over a 5-month period. Samples were taken in triplicate at half-hour intervals and analyzed individually. Hence, every sampling day contains three data points. Error bars on the data points represent the standard error in linear regression in the PME assay.

### PME Storage Stability

Potato PME concentrate was stored “as obtained” from the potato protein factory at Gasselternijveen at temperatures of −28 °C, 4 °C, or at ambient temperature for 4 weeks and analyzed for PME activity.

### Texture Analysis

For texture analysis, gels were prepared using 2 % *w*:*v* of pectin and 5 mM of calcium hydrogen phosphate (final concentrations) with and without a PME dose of 1 unit/g of pectin. Calcium hydrogen phosphate was selected over the corresponding chloride or carbonate to avoid syneresis, uneven setting, and gas formation in the gel matrix [19]. Since untreated pectin samples remained in the dissolved state, a broad and flat probe was necessary to record the viscoelastic properties. These gels were analyzed using a Stable Microsystems texture analyser equipped with a 35-mm diameter cylindrical aluminum probe that was inserted into the gel at a rate of 0.5 mm/s. For every condition, the average of five data points is shown, followed by the standard deviation. Significance of the effect of PME is expressed as *P* value calculated from a two-tailed *T* test.

## Results and Discussion

**Fig. 1:**
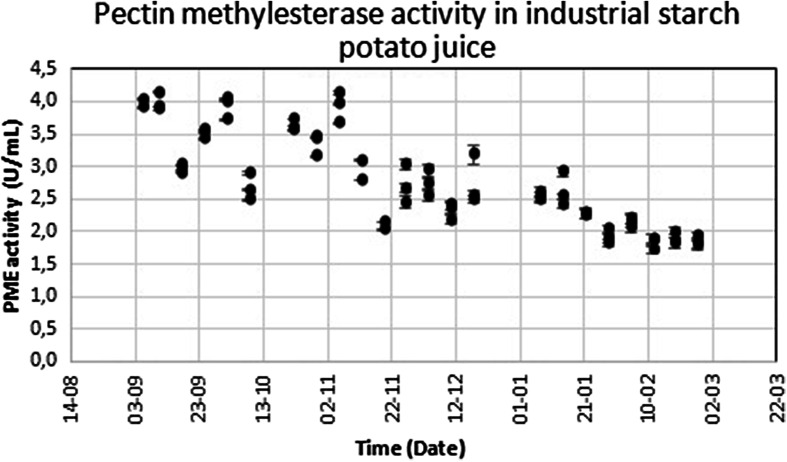
PME activity in industrial destarched potato juice over the course of a harvest campaign. Samples were taken in triplicate, *error bars* show the standard error in the linear regression of the activity determination

**Fig. 2:**
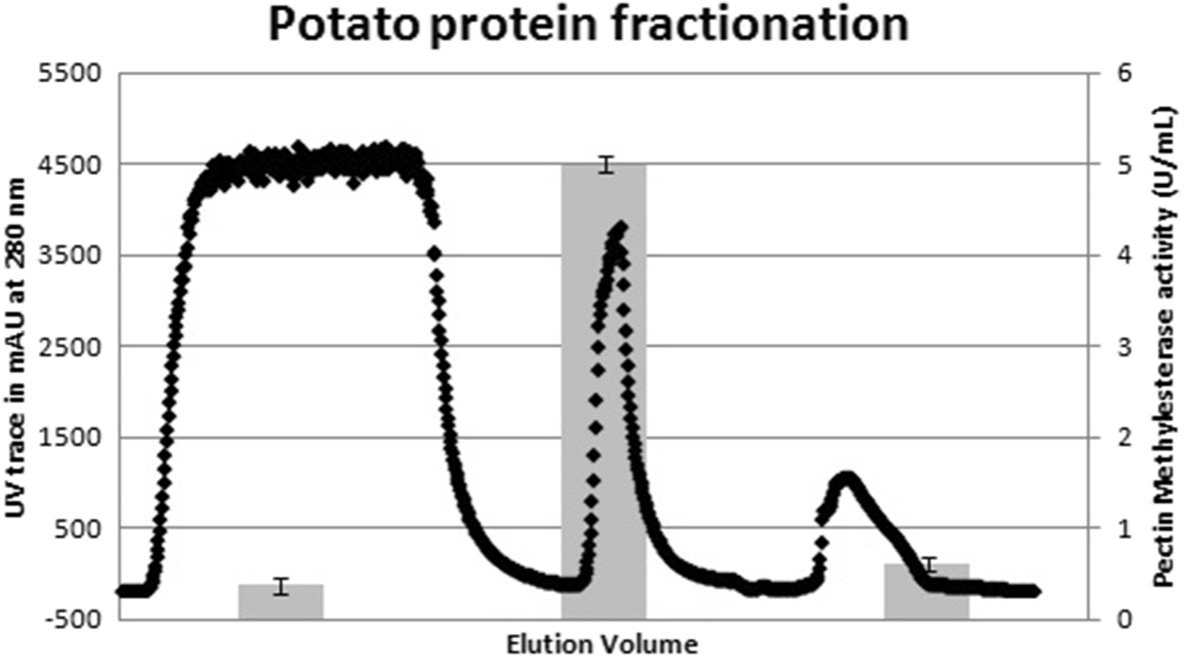
Elution profile of potato protein from destarched potato juice on an expanding bed column as measured by UV spectrometry at 280 nm (*diamonds*, *left axis*), overlaid with PME activity (*bars*, *right axis*). The *leftmost* part shows unbound protein running through the column in the loading step, the *center* shows eluted protein while the *right* part shows uneluted protein that is removed by a cleaning step

Potato juice samples were tested for PME activity to determine the variance in PME activity over the course of the potato campaign. Since the activity was highest in the early months, these represent the most convenient time period for recovering PME from potatoes (Fig. 1). Fractionation of the potato juice showed that the PME activity can be recovered in a single step native protein isolation procedure (Fig. 2), while ultrafiltration was effective in concentrating the activity. In contrast to earlier studies on citrus PME, no organic solvents or precipitation steps were required, allowing for convenient in-line processing.

The PME activity relative to the dry matter content is high compared to the original potato (Table 1). In addition to PME, the fraction contains inert potato protease inhibitors of similar isoelectric points and some buffer salts (data not shown). The PME activity in the final preparation compares positively to that of commercial microbial PMEs (data not shown).

**Table 1:** Concentration procedure for potato PME from tuber pulp

Preparation	% dry matter (*w*:*v*)	% protein (*w*:*v*)	PME activity (U/mL)	Activity/dry matter (U/g)	Specific activity (U/g protein)
Potato pulp	23	1.4	3.6 ± 0.5	16 ± 2	257 ± 36
Potato fruit juice	5.5	10.4	3.4 ± 0.5	62 ± 9	243 ± 36
Potato protein fraction 1	8	7	29.5 ± 1.0	369 ± 13	421 ± 14

**Fig. 3:**
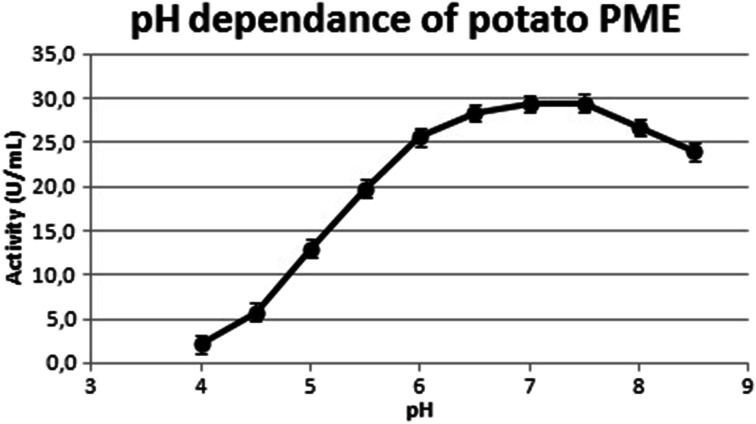
pH dependence of potato PME

**Fig. 4:**
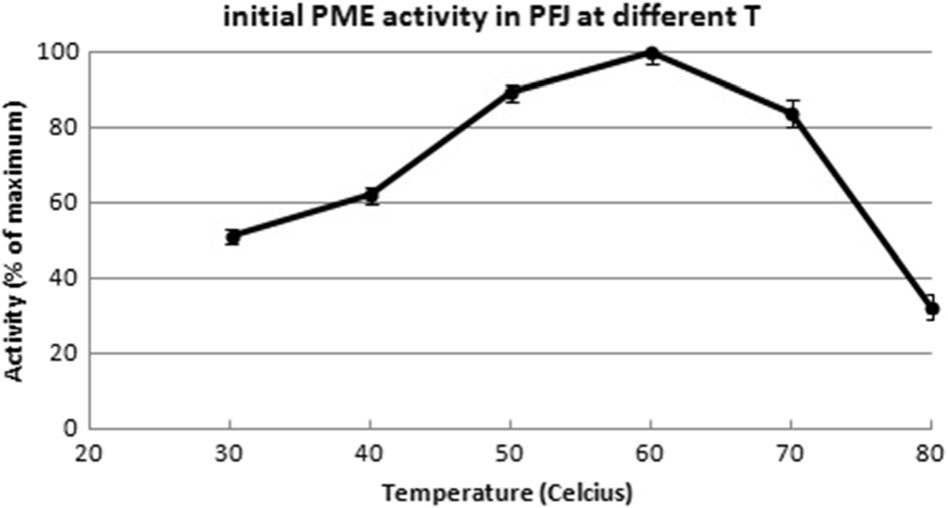
Potato PME activity at varying temperatures expressed as initial reaction rate

The preparation obtained in this way showed pH-dependant PME activity with an optimum at pH 7.5, the profile in good agreement with the data of Puri and coworkers [10], but strikingly different from that reported by Montanez Saenz et al. [9] (Fig 3). The initial PME reaction rate showed a temperature optimum at 60 °C, again similar to Puri but not to Montanez Saenz (Fig 4). Nevertheless, longer exposure to this temperatures resulted in inactivation of the enzyme. Inactivation kinetics for potato PME are known from Anthon and Barrett [17] and the preparation described in this study matches their model (data not shown). Conveniently, this allows for the inactivation of potato PME under normal pasteurizing conditions.

Commercial enzyme preparations that contain PME activity are used in a range of applications, the most common being the maceration of fruits to extract juice [20]. Other applications include the enzymatic firming of fruits and vegetables upon vacuum infusion in combination with calcium and other components as well as the modification of purified pectins to form superior gelling agents and stabilizers for acidified dairy drinks.

Fruit maceration requires a mixture of pectolytic enzymes like pectolyase and polygalacturonase and often contains PME in order to facilitate the latter enzymes action since it requires a demethylated substrate. Although Puri and coworkers reported the presence of low levels of an exopolygalacturonase in potato, a finding later replicated by Anthon and Barrett [17], this enzyme had no effect on pectin viscosity [16]. Dzurova et al. [21] showed the presence of low levels of endopectinases that did reduce pectin viscosity somewhat. These findings may reflect differences in potato cultivar or microbiological contamination between the studies. No studies have so far detected the presence of pectolyase in the potato. Since the preponderance of the literature shows that the potato is naturally low in pectin depolymerizing enzymes, the preparation is not expected to function well in applications where pectin degradation is desired unless it is combined with a different set of enzymes that contains a polygalacturonase.

In contrast, potato PME seems well suitable as a gelling agent for calcium pectin systems and as a stabilizing agent for liquids that contain positively charged particles. This includes the use of PME together with calcium in vacuum infusion since potato PMEs’ broad pH range allows it to function in a variety of fruits and vegetables.

**Fig. 5:**
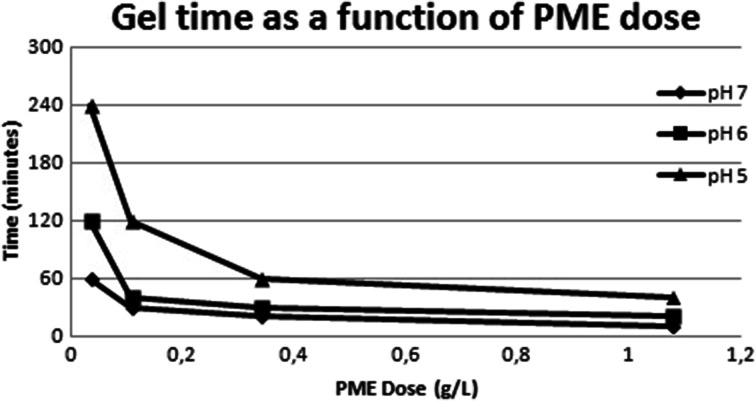
Gel time as a function of PME dose at varying pHs

The effect of potato PME on calcium pectate was tested by visual inspection of self-supporting gels that were generated from calcium pectin solutions, essentially according to the method of O’Brien. Potato PME is active towards citrus pectins of different degrees of esterification (data not shown) and can cause pectin to form a self-supporting gel in the presence of calcium. The ability to induce gel formation depends on the dose of potato PME, the pH of the system, the temperature, and the exposure time (Fig 5).

While the method of O’Brien is fast and convenient, gel properties are more accurately determined by texture analysis [22]. Large deformation analysis was performed to determine gel hardness on PME-induced calcium pectate gels. Upon addition to a 2.0 % *w*:*v* pectin solution containing 5 mM of calcium hydrogen phosphate, the addition of 1 unit of potato PME per gram of pectin induces gel formation over the pH range from 3 and pH 7. The resulting gels differ in firmness but all are significantly stronger than control systems without PME, with the exception of pH 4 where no significant difference was found (Table 2). In agreement with the pH profile, gels prepared at pH 7 are stronger than those prepared at lower pH. The lack of a firming effect at pH 4 likely originates from the decrease in charge density of pectins below pH 4.5. This causes a decreased affinity for calcium ions. Lowering the pH further to 3 allows this effect to be partly counteracted by the formation of hydrogen bonds between protonated carboxyl groups [23].

**Table 2:** Strength of 2.0 % *w*:*v* calcium pectate gels after PME-induced gellation using a dose of 1 unit of PME/g of pectin at 5 mM of CaHPO

pH	Force	Force (PME)	*P*
3	0.1 ± 0.8	4.2 ± 2.4	0.018
4	21 ± 5	16 ± 5	0.308
5	15 ± 3	148 ± 89	0.026
6	10 ± 2	195 ± 37	0.000
7	21 ± 3	220 ± 39	0.000

Keeping the calcium pectate gels at ambient temperature for 2 weeks did not result in syneresis or visible degradation of the gel (data not shown).

**Fig. 6:**
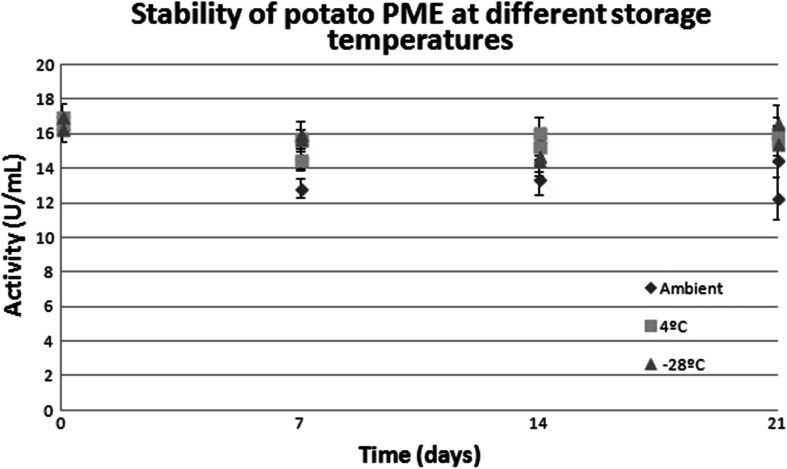
Stability of potato PME at different storage temperatures

The PME preparation can be kept at ambient temperatures without significant losses of activity over a 3-week period (Fig. 6). The addition of stabilizers was not required since the low pH precluded microbial growth.

The overall data shows that potato PME can be conveniently recovered in a form that is suitable for inducing pectin gellation in food systems.

Since the blockwise demethylation pattern of vegetable PME is expected to result in a firmer gel than that of fungal PME, the potato species may cause superior firming in systems where pectin is present in limited amounts. The use of potato PME in fruit firming seems promising and deserves closer attention.

The native potato enzyme recovery technique that is demonstrated in this paper represents a general method of obtaining food and industrial enzymes from vegetable material, in particular from the potato. The use of continuously operating chromatographic systems on vegetable juice streams allows for the rapid and convenient enrichment and purification of valuable enzymatic activities at high volumes in a more sustainable way. In the case of the potato, the most promising enzyme for purification by this method is the potato lipase patatin since purification via EBA technology has been reported [14]. The properties and application of enzymatically active patatin in food systems will be the subject of an upcoming publication.
